# Estimation of linkage disequilibrium levels and allele frequency distribution in crossbred Vrindavani cattle using 50K SNP data

**DOI:** 10.1371/journal.pone.0259572

**Published:** 2021-11-11

**Authors:** Akansha Singh, Amit Kumar, Arnav Mehrotra, Karthikeyan A., Ashwni Kumar Pandey, B. P. Mishra, Triveni Dutt

**Affiliations:** 1 Animal Genetics Division, ICAR–Indian Veterinary Research Institute, Bareilly, Uttar Pradesh, India; 2 Animal Biotechnology Division, ICAR–Indian Veterinary Research Institute, Bareilly, Uttar Pradesh, India; 3 Livestock Production and Management Division, ICAR–Indian Veterinary Research Institute, Bareilly, Uttar Pradesh, India; National Cheng Kung University, TAIWAN

## Abstract

The objective of this study was to calculate the extent and decay of linkage disequilibrium (LD) in 96 crossbred Vrindavani cattle genotyped with Bovine SNP50K Bead Chip. After filtering, 43,821 SNPs were retained for final analysis, across 2500.3 Mb of autosome. A significant percentage of SNPs was having minor allele frequency of less than 0.20. The extent of LD between autosomal SNPs up to 10 Mb apart across the genome was measured using r^2^ statistic. The mean r^2^ value was 0.43, if pairwise distance of marker was less than10 kb and it decreased further to 0.21 for 25–50 kb markers distance. Further, the effect of minor allele frequency and sample size on LD estimate was investigated. The LD value decreased with the increase in inter-marker distance, and increased with the increase of minor allelic frequency. The estimated inbreeding coefficient and effective population size were 0.04, and 46 for present generation, which indicated small and unstable population of Vrindavani cattle. These findings suggested that a denser or breed specific SNP panel would be required to cover all genome of Vrindavani cattle for genome wide association studies (GWAS).

## Introduction

Linkage disequilibrium (LD), defined as the degree of non-random association of allele between loci or correlation between genotypes of markers, is an important concept in the understanding of gene mapping and its application in genetic studies. The LD between markers provides insight in exploring the level of diversity between different breeds, inferring the frequency of recombination events, investigating the change in effective population size across generations, and identifying genomic regions to improve economically important traits [1–3]. The pattern of LD is mainly determined by the physical distance between markers, although several other demographic and evolutionary factors including population stratification, inbreeding, effective population size, genetic bottleneck, genetic drift, migration, mutation, selection, and recombination rate may also influence the extent and pattern of LD [4–6].

The declining cost of high throughput genotyping provides a new opportunity to explore genome-scale studies, especially Genome Wide Association Studies (GWAS) and Genomic Selection (GS). The accuracy and precision of the genomic studies rely on the level and pattern of linkage disequilibrium (LD) between markers across genome [7]. The LD between markers has been studied in the genome of several taurine and indicine cattle breeds [2, 8]. Results from their study revealed that moderate LD (*r*^2^ = 0.20) were extended to <100 kb, which indicated that 50K SNPs captures most of the LD information necessary for GWAS in taurine breeds [1, 2]. However, the extent for of LD (*r*^2^ = 0.20 to 0.34) was observed lower (<30kb) within indicine cattle, which indicated use of High density chip for genomic studies in these cattle [8]. The reduced extent of LD detected in indicine cattle breeds can be attributed to the ascertainment bias of the SNP Chips.

Vrindavani is four breed synthetic crossbred cattle strain developed in India by crossing between Bos *taurus* cattle (Holstein, Brown Swiss, Jersey) and Bos *indicus* cattle (Hariana) [9]. Recently, the ROH fragments, haplotype blocks, inbreeding coefficient and effective population size in Vrindavani cattle has been evaluated [10]. Further, the admixture pattern and signatures of selection in Vrindavani was identified using 50K SNP chip [9]. However, the extent and pattern of genome-wide LD using 50K SNP Chip in Vrindavani cattle remains unexplored till date. This study was indented to build on the previous work of genetic characterization [9], and gives insight to estimate marker density for genomic studies in Vrindavani cattle. The objective of present study was to investigate allelic frequency distribution, extent of linkage disequilibrium level (r^2^) and estimate the effective population size in crossbred Vrindavani cattle using a 50K Bovine chip.

## Material and methods

### Animal sample and genotype quality control

A total of 96 female Vrindavani crossbred cattle were selected from ICAR-Indian Veterinary Research Institute (IVRI), Izatnagar, Bareilly (Uttar Pradesh), India. The experiment was approved by the Institutional Animal Ethics Committee (IAEC) of ICAR-Indian Veterinary Research Institute (IVRI). DNA was extracted from blood samples and genotypic data were generated using the Bovine 50K SNP Bead Chip v2 (Agri Genome Labs Pvt. Ltd., India).

The HiScan images and genotypes were analysed using Genome Studio software (Illumina). The genotyping rate was 0.99, and a total of 53,218 SNPs were identified across the genome at a mean distance of 37.4 kb between markers. Quality control (QC) was performed using the PLINK v1.9 software [11]. The SNPs with call rate of less than 95%, MAF of less than 0.02 and HWE less than 10E x 10^−05^ were removed. Only SNPs that were uniquely mapped to autosomes were included in the analysis. A total of 43,821 markers were retained for further analysis. The length of chromosome (Mb), number of markers on individual autosome and the mean distance between markers were estimated using R software [12].

### Minor allele frequency

The MAF for all autosomal SNPs was calculated using PLINK v1.07 as default settings using command—file data—freq [11]. The distribution of allele frequencies across different chromosome was analysed, and proportion of SNPs in different frequency category (0.02–0.10, 0.10–0.20, 0.20–0.30, 0.30–0.40, and 0.40–0.50) were plotted using R software [12].

### Inbreeding coefficient and effective population size

Inbreeding coefficient (F) was calculated as a function of the expected and observed homozygote difference using PLINK v1.07 [11]. The equation is given as follow:

$$F_{i}=\frac{O_{i}-E_{i}}{L_{i}-E_{i}}$$

where *F*_*i*_ is the estimated inbreeding coefficient of the *i*^*ih*^ animal; *O*_*i*_ is the number of observed homozygous loci in the *i*^*ih*^ animal, *E*_*i*_ is the number of expected homozygous loci and *L*_*i*_ is the number of genotyped autosomal loci.

The Effective population size (Ne) was estimated using the SNeP tool [13], based on the relationship between *N*_*e*_, linkage disequilibrium represented by r^2^, and recombination rate (*c*). This is given by:

$$T_{t}=\left(4\int {\left(c_{t})\right)}^{-1}\right)(E[{{(r}_{adj}^{2}|c_{t}]}^{-1}-\alpha )$$

where, N_T_ is the effective population size t generations ago, c_t_ is the recombination rate, r^2^_adj_ is the LD value adjusted for sample size, and *α* is he correction for occurrence of mutation.

### Linkage disequilibrium

The square of the correlation coefficient between two loci (r^2^) was used to evaluate the extent of LD measure, as r^2^ was considered is considered as more robust and not influenced by change in allele frequency and population size [14]. The r^2^ estimate is an important estimator to measure loci required for genome wide association study and QTL mapping [8]. The equation for LD (r^2^) estimate is represented as follows:

$$r^{2}=\frac{{\left(P_{11}P_{12}-P_{12}P_{21}\right)}^{2}}{P_{A1}P_{A2}P_{B1}P_{B2}}$$

where, P_A1_, P_A2_, P_B1_ and P_B2_ are frequencies of each allele at loci A and B, and P_11_, P_12_, P_21_ and P_22_ are the frequencies of haplotype A_1_B_1_, A_1_B_2_, A_2_B_1_, A_2_B_2_, respectively.

The r^2^ between SNP pairs with physical distances between 0 to 10 Mb for all autosome was used to estimate the extent and pattern of LD using the default command of PLINK v1.07 using command–ld-snp-list mysnplist–ld-window-kb 10000 –ld-window 99999 –ld-window r2 0 [11]. The decay of LD value, were then calculated for distances of pair-wise SNPs were interval into eight categories (0–10 kb, 10–25 kb, 25–50 kb, 50–100 kb, 100–500 kb, 0.5–1 Mb, 1–5 Mb and 5–10 Mb) along the first 10 Mb of each autosome. LD was also estimated as the mean r^2^ value for each autosomal chromosome according to these intervals and plotted against distance range.

Further the effect of minimum allelic frequency (MAF) and sample size on the extent of LD was investigated. The LD was calculated as previously described with four different MAF thresholds (0.05, 0.10, 0.15 and 0.2) for physical distance of 10 Mb of genome. In order to determine the effect of sample size on LD(r^2^), five different subset of population (N = 10, 25, 50, 75 and, 90) were randomly selected from the total population and the extent of LD was explored for each sub-population. To visualize the effects of MAF and sample size on the genome-wide LD (r^2^) levels was also plotted accordingly.

## Results

### Marker statistics

After quality control, a total 43,821 autosomal SNPs were available for downstream analysis. About 6734 SNPs having MAF less than 0.02 were removed, and 723 SNPs were removed on the basis Hardy-Weinberg equilibrium and call rate threshold criteria. The SNPs retained after quality control spanned across total of 2500.30 Mb of region of Vrindavani genome, with a mean chromosome length of 86.21Mb. The BTA1(158.03Mb) was found to be longest, while the BTA25 (42.80Mb) was shortest across the genome. The distribution of SNPs was proportional to length of chromosome; the highest number of SNPs were on BTA1 (2798), and the lowest on BTA25 (792). The average distance between SNPs was 57.24 kb, where the longest distance between SNPs was 3.26 Mb on BTA10 and shortest distance was 0.01 kb on BTA15. The descriptive statistical results of the SNP marker for each autosome are shown in Table 1.

**Table 1:** Summary of the SNP markers analyzed and minor allele frequency for each autosomal chromosome (BTA).

BTA	Length (Mb)	Number of SNP	Average SNP interval (Mb)	Longest SNP Interval (Mb)	Shortest SNP interval (kb)	MAF
1	158.026	2798	0.057	0.759	0.131	0.267
2	136.662	2249	0.061	0.991	0.075	0.260
3	121.144	2117	0.057	0.807	0.108	0.263
4	120.281	2031	0.059	0.485	0.365	0.256
5	121.079	1859	0.065	1.027	0.479	0.259
6	119.014	2513	0.047	1.602	0.089	0.277
7	112.384	2170	0.052	1.124	0.596	0.279
8	112.908	1994	0.056	0.482	0.084	0.258
9	105.464	1754	0.060	0.667	0.449	0.260
10	104.164	1981	0.053	3.259	0.284	0.261
11	107.178	1840	0.058	0.717	0.886	0.266
12	90.819	1347	0.067	2.198	0.237	0.261
13	83.863	1472	0.057	0.676	0.065	0.264
14	83.153	1450	0.057	0.556	0.108	0.256
15	84.445	1430	0.059	0.851	0.010	0.258
16	81.249	1392	0.058	1.066	0.178	0.261
17	74.887	1320	0.057	0.779	0.778	0.254
18	65.401	1122	0.058	0.967	0.493	0.271
19	63.541	1178	0.054	0.545	0.401	0.270
20	71.595	1398	0.051	0.666	0.466	0.265
21	71.098	1220	0.058	0.764	0.485	0.263
22	61.216	1042	0.059	0.523	0.246	0.265
23	52.096	981	0.053	0.402	0.785	0.267
24	62.102	1030	0.060	0.431	0.095	0.257
25	42.804	792	0.054	0.283	0.784	0.275
26	51.046	900	0.057	0.342	0.281	0.263
27	45.332	801	0.057	0.566	0.151	0.254
28	46.183	785	0.059	0.400	0.675	0.260
29	51.102	885	0.060	1.507	1.762	0.274

### Minor allele frequency

**Fig 1:**
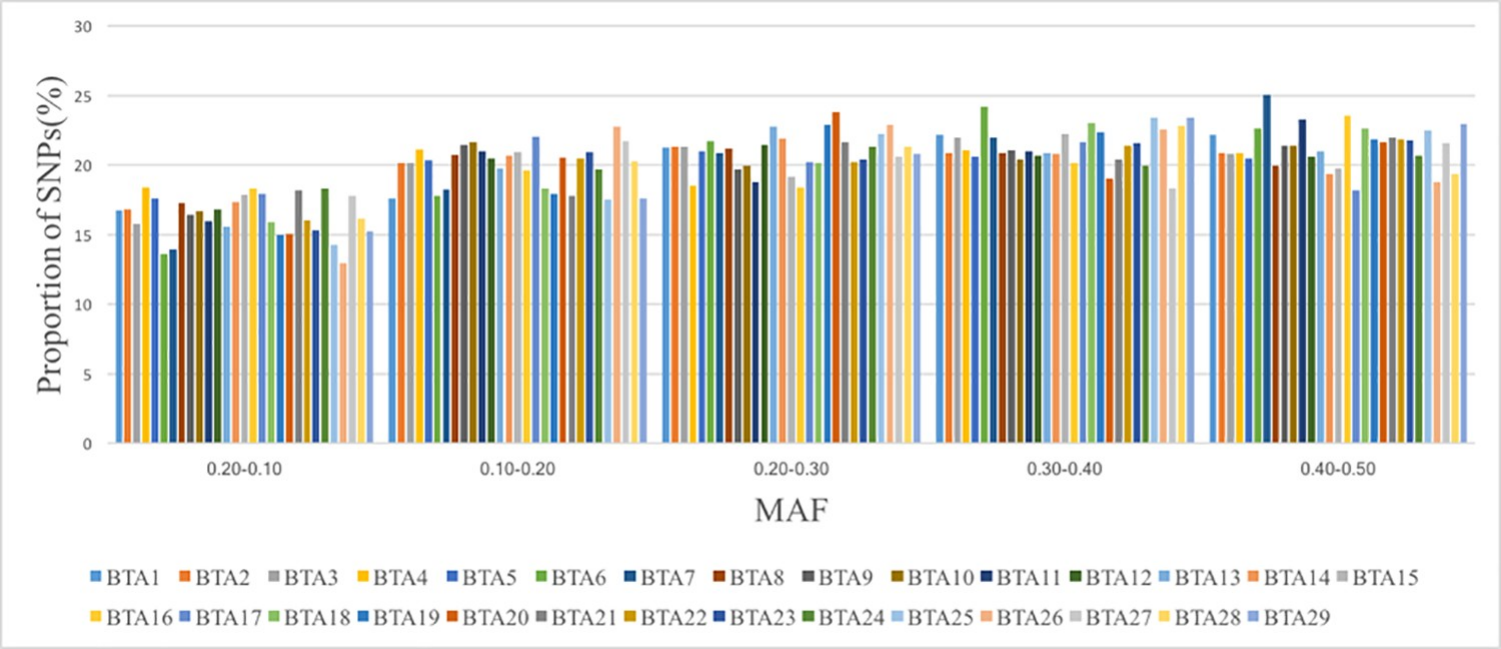
Mean proportion of SNPs for various minor allele frequencies (MAF) calculated for each autosomal chromosome.

The mean minor allele frequency (MAF) across all autosomes was 0.26 (Table 1). After filtering, 36% of the SNPs (15,805) the MAF was lower than 0.20. The proportion of SNPs on each chromosome at different MAF threshold is presented in Fig 1. While the MAF was lower than 0.10 (0.2–0.10), the chromosome BTA 4, BTA16, BTA21 and BTA24 had a higher SNP proportion, whereas BTA6, BTA7 and BTA27 presented a lower proportion of SNPs.

### Effective population size and inbreeding coefficient

The effective population size was estimated over the past 50 generations from the average r^2^ (Table 2). The estimate of Ne decreased from 245 (50 generations ago) to 46 (5 generations ago), which reflect a declining trend in N_e_ for Vrindavani. The estimate of mean inbreeding coefficient (F) was 0.04.

**Table 2:** Effective population size (Ne) over generations based on linkage disequilibrium calculations.

Generations Ago	Ne	Distance	r^2^	r^2^(SD)
5	46	9426855	0.0567	0.0758
6	49	8342446	0.0598	0.0796
7	53	7366372	0.0627	0.0830
7	57	6489771	0.0654	0.0865
9	61	5709032	0.0685	0.0896
10	67	5013219	0.0712	0.0930
11	73	4397475	0.0742	0.0966
13	79	3855051	0.0769	0.0996
15	86	3381675	0.0803	0.1032
17	96	2969308	0.0821	0.1055
19	106	2612771	0.0837	0.1071
21	116	2307237	0.0866	0.1116
24	128	2047260	0.0880	0.1125
27	141	1828020	0.0893	0.1139
30	154	1645198	0.0907	0.1149
33	166	1494458	0.0922	0.1179
36	178	1372177	0.0935	0.1184
39	188	1273826	0.0951	0.1197
42	199	1196571	0.0956	0.1215
44	205	1136869	0.0974	0.1231
46	217	1092011	0.0959	0.1217
47	221	1059183	0.0971	0.1225
48	228	1036170	0.0961	0.1217
49	223	1020495	0.0993	0.1256
50	245	1001866	0.0930	0.1141

### Extent of LD across the genome

The level of LD among SNPs was estimated by r^2^ method. The free recombination generally occurs at a physical distance of more than 10 Mb [15] thus, the range between SNPs was set at 0–10 Mb to estimate LD between SNP markers. In order to consider all possible pairs of SNPs with a distance of less than or equal to 10 Mb, combination pairs of 7,378,918 SNPs were obtained across autosomes to estimate LD. The mean values of r^2^ of markers for distance of 10 Mb was 0.07. The mean LD (r^2^) for physical distance intervals is presented in Table 3. The autosomes BTA16, BTA24, and BTA 20 had higher level of r^2^. For the physical distance of less than 10 kb, the average r^2^ was 0.43 and it decreased to 0.21 for the distances of 25 to 50 kb. Results revealed the decline in mean r^2^ value with the increase in physical distance between markers.

**Table 3:** Statistical summary of linkage disequilibrium (r^2^) over various distances.

Distance	Number of SNP pairs	Mean r^2^
0–10 kb	2926	0.43
10–25 kb	9947	0.26
25–50 kb	27339	0.21
50–100 kb	49312	0.16
100–500 kb	349058	0.11
0.5–1 Mb	401738	0.09
1–5 Mb	3034419	0.08
5–10 Mb	3504174	0.06

**Fig 2:**
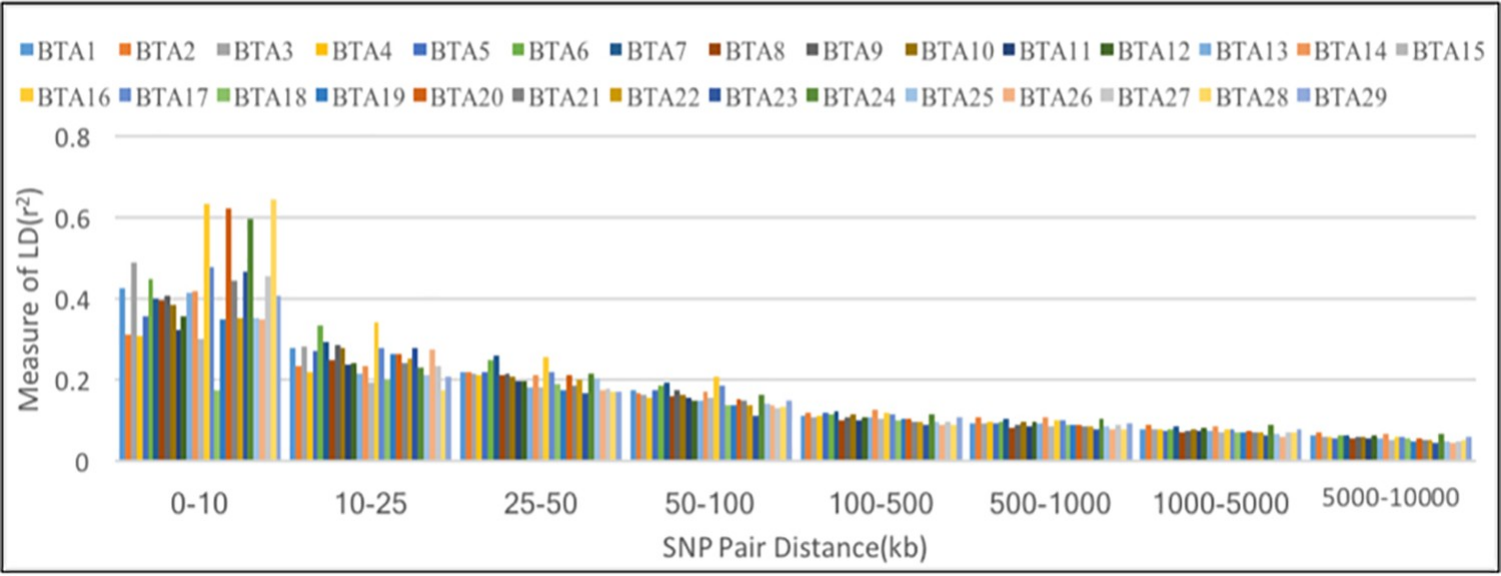
The mean *r*^2^ as distance between pairs of SNP up to 10 Mb for the genome across autosomes.

The extent of LD (r^2^) was found different across each autosomes according to the physical distance. The mean r^2^ was estimated for markers physical distance separated by intervals of 0–10 kb, 10–25 kb, 50-100kb, 100-500kb, 0.5-1Mb, 1-5Mb, and 5–10 Mb per autosome (Table 4). The mean r^2^ revealed smaller reduction across different autosomes, when the physical distance between markers exceeded 100 kb (Fig 2).

**Table 4:** Statistical information for mean r^2^ as physical distance between SNP pairs up to 10 Mb for the genome.

BTA	0-10kb	10-25kb	25-50kb	50-100kb	100-500kb	0.5-1Mb	1-5Mb	5-10Mb
1	0.424±0.041	0.277±0.013	0.216±0.006	0.175±0.004	0.110±0.001	0.091±0.001	0.079±0.001	0.064±0.000
2	0.310±0.036	0.231±0.012	0.216±0.007	0.165±0.004	0.118±0.001	0.106±0.001	0.089±0.000	0.070±0.000
3	0.489±0.027	0.279±0.012	0.214±0.006	0.164±0.004	0.105±0.001	0.092±0.001	0.076±0.000	0.057±0.000
4	0.306±0.142	0.218±0.016	0.209±0.007	0.156±0.004	0.109±0.001	0.095±0.001	0.079±0.000	0.060±0.000
5	0.355±0.034	0.269±0.014	0.218±0.008	0.172±0.005	0.118±0.001	0.091±0.001	0.073±0.000	0.055±0.000
6	0.448±0.025	0.331±0.012	0.247±0.006	0.183±0.004	0.114±0.001	0.096±0.001	0.079±0.000	0.064±0.000
7	0.399±0.027	0.292±0.013	0.257±0.007	0.191±0.004	0.122±0.001	0.103±0.001	0.085±0.000	0.061±0.000
8	0.397±0.109	0.249±0.018	0.209±0.008	0.157±0.004	0.099±0.001	0.082±0.001	0.070±0.000	0.055±0.000
9	0.408±0.025	0.286±0.014	0.214±0.007	0.172±0.004	0.106±0.001	0.087±0.001	0.075±0.000	0.059±0.000
10	0.386±0.013	0.277±0.008	0.205±0.004	0.164±0.003	0.115±0.001	0.097±0.001	0.078±0.000	0.058±0.000
11	0.323±0.112	0.235±0.015	0.196±0.008	0.154±0.004	0.100±0.001	0.084±0.001	0.072±0.000	0.056±0.000
12	0.353±0.126	0.239±0.019	0.195±0.009	0.146±0.005	0.107±0.001	0.094±0.001	0.082±0.000	0.063±0.000
13	0.413±0.089	0.215±0.018	0.180±0.008	0.149±0.005	0.106±0.001	0.093±0.001	0.073±0.000	0.054±0.000
14	0.418±0.148	0.232±0.019	0.211±0.009	0.169±0.005	0.124±0.001	0.107±0.001	0.086±0.000	0.066±0.000
15	0.298±0.025	0.192±0.011	0.180±0.007	0.154±0.005	0.103±0.001	0.083±0.001	0.071±0.000	0.052±0.000
16	0.631±0.026	0.341±0.016	0.253±0.009	0.208±0.006	0.119±0.001	0.099±0.001	0.078±0.000	0.058±0.000
17	0.476±0.043	0.277±0.017	0.219±0.009	0.185±0.006	0.113±0.001	0.101±0.001	0.076±0.000	0.058±0.000
18	0.172±0.024	0.198±0.015	0.188±0.008	0.138±0.005	0.100±0.001	0.088±0.001	0.071±0.000	0.054±0.000
19	0.349±0.025	0.264±0.015	0.174±0.007	0.136±0.004	0.102±0.001	0.087±0.001	0.068±0.000	0.049±0.000
20	0.622±0.020	0.261±0.013	0.209±0.007	0.153±0.004	0.102±0.001	0.087±0.001	0.074±0.000	0.056±0.000
21	0.442±0.028	0.239±0.014	0.185±0.007	0.148±0.004	0.097±0.001	0.085±0.001	0.071±0.000	0.052±0.000
22	0.352±0.109	0.252±0.025	0.198±0.010	0.135±0.005	0.097±0.001	0.084±0.001	0.068±0.000	0.050±0.000
23	0.465±0.028	0.278±0.015	0.167±0.007	0.111±0.004	0.088±0.001	0.077±0.001	0.062±0.000	0.044±0.000
24	0.596±0.233	0.229±0.021	0.214±0.012	0.162±0.006	0.116±0.002	0.102±0.001	0.088±0.000	0.067±0.000
25	0.352±0.086	0.211±0.021	0.203±0.012	0.140±0.006	0.097±0.002	0.086±0.001	0.067±0.000	0.048±0.000
26	0.349±0.209	0.272±0.030	0.173±0.011	0.137±0.006	0.090±0.001	0.076±0.001	0.060±0.000	0.044±0.000
27	0.456±0.041	0.234±0.019	0.178±0.010	0.129±0.006	0.097±0.002	0.087±0.001	0.068±0.000	0.049±0.000
28	0.643±0.140	0.174±0.020	0.169±0.011	0.131±0.006	0.088±0.002	0.079±0.001	0.069±0.000	0.052±0.000
29	0.408±0.134	0.205±0.022	0.168±0.010	0.147±0.006	0.106±0.002	0.091±0.001	0.077±0.000	0.057±0.000

### MAF and LD estimates

**Fig 3:**
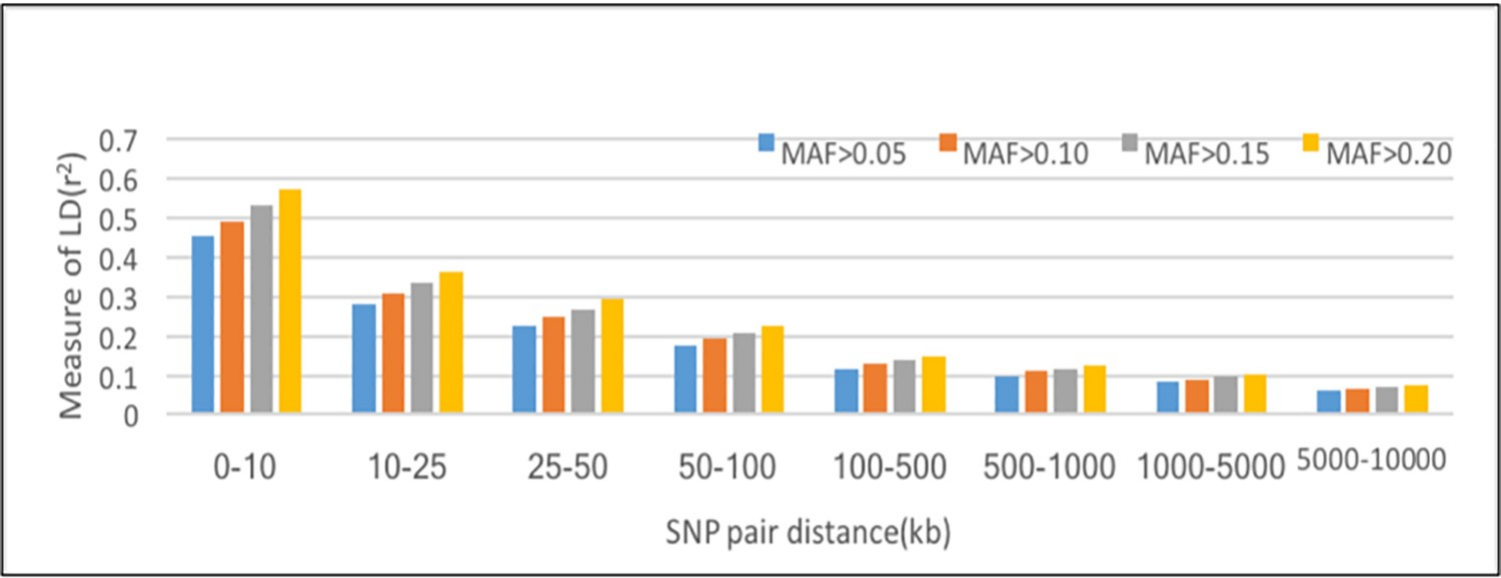
Mean r^2^ estimates at different physical distances for different minor allelic frequency (MAF) thresholds.

The effect of MAF on LD extent was estimated using four different threshold level of MAF: >0.05, >0.10, >0.15 and 0.20 for physical distances up to 100kb between the SNP pairs (Fig 3). Results showed significant effect of MAF threshold on the average *r*^*2*^, particularly for less distances. The r^2^ between SNPs was smaller when the threshold of MAF was low (0.05), and increases significantly at higher MAF threshold. The value of mean r^2^ ranged from 0.45 to 0.06 for MAF > 0.05, 0.49 to 0.07 for MAF> 0.10, 0.53 to 0.07 for MAF> 0.15, and 0.57 to 0.08 for MAF> 0.20, respectively.

### Sample size and LD estimates

**Fig 4:**
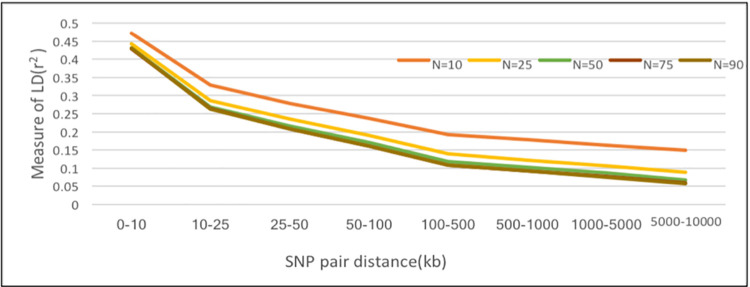
Mean r^2^ estimates at different physical distances for different sample size.

In this investigation, sample size (n) of 10, 25, 50, 75, and 90 were selected at random from the total population to assess the effect of sample size on LD (r^2^) extent (Fig 4). The average r^2^ was greater for smaller sample size, and this trend was more evident when physical distance intervals were large (more than 50kb). These findings indicated that the size of samples should be at least 50 animals for precise estimation of r^2^.

## Discussion

After quality control, a total of 43,821 autosomal SNPs retained, which were distributed across 2500.30 Mb region of Vrindavani genome. The average MAF value was 0.26, which is comparable with the value reported for different cattle breeds [8]. The distribution of MAF directly influenced the LD, as low MAF could correspond to greater difference in the frequency of allelic pairs, resulted in underestimation of LD [16]. Therefore, four different level of MAF were selected to estimate the effect of MAF on LD extent. The result showed that the average LD (r^2^) between SNPs was high, when the threshold of MAF was high (>0.20), particularly at smaller distance. Similar effect of allele frequency on r^2^ estimate was also observed in other cattle breeds [17, 18], suggesting that the 50K chip can be used for genetic studies in crossbred Vrindavani cattle.

The LD between markers was estimated using r^2^ method as it is less influenced by low allele frequency [5] and small sample size [15]. At inter-marker distance of 10kb, the mean r^2^ value was 0.43 which was lower as compared to the LD estimates previously reported for taurine breed, Angus (0.46) and Hereford (0.49), but higher than the value reported for indicine breeds of Brahman (0.25), Nellore (0.27) cattle [2, 3]. The mean LD between adjacent value was estimated for different intervals across autosomes. The mean r^2^ for was highest for BTA16 and BTA24 and lowest BTA18 and BTA15. However, no relationship was observed between chromosomal size and r^2^ estimate [19]. To explore the dependency of sample size on the extent of LD (r^2^), different sample size was used to calculate r^2^. Khatkar et al. [18] has reported that small sample size leads to overestimation of LD. In the current study, a minimal sample size of 55 animal had no influence on r^2^, which is consistent with the previous study [19].

To analyse the LD decay, the physical distance between markers were classified in different interval. The results showed rapid decay of r^2^ at distance more than 100kb for all autosomes. Moreover, the LD estimate decreased from 0.43 to 0.21 at 10kb and 50kb marker distance, respectively. Similar level of LD estimates at 40-60kb have been reported for taurine cattle breeds [18, 20]. However, low level of LD estimate had previously been reported in indicine cattle, which may be attributed to ascertainment bias of SNPs in the current chip [2].

The reliability and accuracy of genomic association studies and genomic prediction is dependent on the amount of LD between marker. Mckay et al. [1] reported that a total of 28,700 (2.87GB/100kb at r^2^ = 0.2, where 2.87GB is the bovine genome size) SNPs were required to capture LD information for genomic studies. In our study, the LD (r^2^) estimate was 0.21 at distance of 50 kb between SNPs. This indicates that a minimum of 57,400 (2.87 GB/ 50kb at r^2^ = 0.2) SNPs is required for genomic association studies in Vrindavani cattle. Previous studies have also indicated the requirement of large number of SNPs to cover the genome for genomic studies when analysing data from indicine, crossbred and multi-breed populations [2, 8]. Our data suggest that a higher density SNP array were required for reliable genome-wide association mapping and genomic prediction in crossbred Vrindavani population.

For better understanding of the population structure, the inbreeding coefficient and the effective population size were estimated. The inbreeding coefficient was 0.04, which was comparable to previously reported 3% and 6% inbreeding coefficient in Vrindavani population using ROH and F_HOM_ analysis [10]. The current study observed decrease in effective population size from 5 generations ago to 50 generations ago. Further, the estimate of N_e_ was 40 in Vrindavani, and a decreasing trend of N_e_ was reported for over five generations. The FAO has recommended N_e_ should be at least 50 for maintaining proper breeding plan. In the current study, 5 generations ago the estimated N_e_ (46), was below the recommended number (50) of FAO. The low N_e_ estimate may be attributed to the small well protected population size and fluctuating breed ratio in the admixed Vrindavani population [10].

## Conclusion

This study reported the magnitude of LD between markers crossbred Vrindavani cattle using 50k Bovine Chip. The estimated r^2^> 0.2 extended up to 50 kb indicates requirement of high density SNP panel for precise and accurate estimation of whole genome association studies in Vrindavani cattle. Furthermore, declining trend of N_e_ estimates was observed in Vrindavani population indicates the requirement of breeding plan that could maintain the sufficiently large Ne. However, further confirmatory investigating for the extent of LD and effective population is required in larger population using high density array of SNPs.
